# Enhancing cross-cultural well-being: a mixed methods study on critical thinking, cultural intelligence, and eudaimonic well-being in arts students’ cultural identity development

**DOI:** 10.3389/fpsyg.2024.1425929

**Published:** 2024-11-13

**Authors:** Yue Peng

**Affiliations:** School of Arts and Humanities, Guangzhou Academy of Fine Arts, Guangzhou, China

**Keywords:** psychological well-being, critical thinking, cultural identity development, cultural intelligence, arts students

## Abstract

**Introduction:**

This research explores the role of critical thinking and cultural intelligence in psychological well-being through the lens of cultural identity development. It aims to understand how individuals think critically and navigate different cultural challenges that influence their psychological wellbeing.

**Methods:**

The quantitative analysis utilizes various scales to elucidate the correlation between critical thinking beliefs, cultural intelligence, and well-being. The qualitative exploration employing NVivo 20.0 uncovers the interplay between critical thinking, cultural intelligence, and well-being in the construction of cultural identity and psychological selfhood.

**Results:**

The research results show a significant positive correlation between critical thinking, cultural intelligence, and psychological well-being. Themes such as well-being, cultural intelligence, critical thinking, and facing challenges illustrate how individuals navigate obstacles to achieve a meaningful and purposeful life. The findings from both the quantitative and qualitative analyses provide a comprehensive understanding of how critical thinking, cultural intelligence, and well-being intersect and influence individuals’ perceptions of themselves and their cultural identities.

**Discussion:**

The research results suggest that developing critical thinking and cultural intelligence positively impacts individuals’ psychological well-being in cultural identity development. This can lead to greater belonging and acceptance in diverse cultural settings.

## 1 Introduction

Psychological well-being is a concept of overall life satisfaction. Ryff’s (2014) multidimensional model is a significant framework for understanding psychological well-being. It focuses on the significant dimensions for achieving well-being, including self-acceptance, purpose in life, personal growth, positive relationships, autonomy, and environmental mastery. This perspective emphasizes pursuing a meaningful and fulfilling life, highlighting self-realization, personal growth, and the pursuit of virtue and excellence. Besides, Ryff (2023a) also proposed that cultures are significant for meaning-making while achieving psychological well-being. According to Ryff (2023b), meaning-making is crucial at individual and societal levels. At the intrapersonal level, it is essential to acknowledge and process a wide range of emotions in comprehending social changes; at the interpersonal level, history, philosophy, and the arts play fundamental roles in developing the individual ability “to understand, to care, and to act.” Considering that, cultures play an important role in promoting awareness, perception, and comprehension of social context, providing insight into how life’s purpose should be constructed. From the perspective of psychological well-being, researchers might study various topics, such as sociodemographic factors, psychosocial influences, developmental changes over the lifespan, and differences in biological and health outcomes (Waterman, 2008). However, individuals’ subjective initiative significantly impacts their destinies, behaviors, and decisions throughout their lifespan. In today’s globalized world, individuals are often exposed to the influences of multiple cultures simultaneously, both externally and internally. Therefore, it is essential to employ a holistic approach when exploring cultures. Examining the cultural identity development process is a new perspective for understanding how individuals negotiate, adjust, and adapt to life challenges to achieve well-being. Accordingly, three research questions are addressed in this research:

RQ 1: How do critical thinking belief and cultural intelligence interact to impact individuals’ eudaimonic well-being?

RQ2: How do critical thinking, cultural intelligence, and psychological selfhood affect individuals’ well-being?

RQ3: How do critical thinking, cultural intelligence, and eudaimonic well-being contribute to the development of individuals’ cultural identities?

Well-being is deeply intertwined with personal feelings and interpretations of life experiences. This research, comprising quantitative and qualitative studies, highlights the universal aspects of well-being and brings attention to the culturally specific elements that influence how well-being is experienced, expressed, and constructed. The quantitative analysis will provide measurable insights into the relationships between critical thinking, cultural intelligence, and eudaimonic well-being. The qualitative analysis will focus on individual experiences and narratives navigating the challenges of identity development, which are not captured by psychological quantitative measurement. By exploring the complex ways in which critical thinking, cultural intelligence, and eudaimonic well-being contribute to personal growth, this study aims to offer a more comprehensive understanding of cultural identity development.

## 2 Literature review

### 2.1 Psychological well-being and cultural identity development

Psychological well-being is an expanded concept encompassing various elements, including—according to Ryff (1989, 2014)—self-acceptance, positive relationships with others, autonomy, environmental mastery, purpose in life, and personal growth. These elements contribute to mental health, self-actualization, optimal functioning, and maturity by emphasizing the importance of positive attitudes toward oneself, warm relationships, independence, control over one’s environment, a sense of purpose, and continuous personal development. Overall, these dimensions provide a comprehensive fundamental framework for understanding and measuring psychological well-being that focuses on pursuing a meaningful and fulfilling life. As Ryff (2023a) states, psychological well-being should be accompanied by the ability to overcome challenges in real life. Some studies attempt to discover how an individual’s character impacts their happiness, life satisfaction, and emotional fulfillment. There are some researches focus on the relationship between various dimensions of subjective well-being in different age groups, contexts, and spaces between personalities (Albuquerque et al., 2012; Le Moglie et al., 2015; Stead and Bibby, 2017; Etxeberria et al., 2019; Gilberto et al., 2020; Barthelmäs and Keller, 2021; Fan et al., 2022; Kohút et al., 2022; Mussone and Changizi, 2023; Schneider et al., 2024). The others focus on the relationship with emotional intelligence (Schutte and Malouff, 2011; Bhullar et al., 2013; Vergara et al., 2015; Sánchez-Álvarez et al., 2016; Aloia and Brecht, 2017; Martínez-marín and Martínez, 2019; Alshakhsi et al., 2022; Martinez-Marin and Martínez, 2022; Mancini et al., 2024). However, the issues about exploring well-being from the cognitive and cultural perspective is rarely discussed. Thus, this research attempts to discover the interplay of these two aspects of psychological well-being.

Identity is multi-dimensional as it encompasses a combination of social and psychological attributes. Moran (2009) divided measurements of identity into personal (i.e., behaviors, emotions, beliefs, knowledge, and values) and social (i.e., racial, ethnic, and gender) dimensions. Most of these tools evaluate the degree to which an individual has achieved a particular level of identity along a particular dimension (e.g., ethnic identity). Identity development and formulation appear complicated. Some studies provide evidence that the construction of one’s identity is a continuous undertaking that transcends personal, social, and cultural boundaries, such as through various life stages (Marcia, 2002; Duerden et al., 2009; Pnevmatikos et al., 2010; Boskey, 2014; Green et al., 2016) and cultures (Germain, 2004; Walters and Auton-Cuff, 2009; Nguyen et al., 2012; Arneaud et al., 2016; du Plessis and Naudé, 2017; Renn, 2020). Cultural identity is a complex concept involving individual and social dimensions. Developing cultural identity is a multifaceted process involving various factors that shape an individual’s sense of self. Culture, philosophy, history, and the arts can activate awareness, prompt social change, and transform suffering into personal growth (Ryff 2023a). According to Mrazek et al. (2015), cultural differences in self-construal style shape individuals’ views of the self in both the mind and brain. Cultural beliefs significantly impact individuals’ self-awareness and self-evaluation (Mrazek et al., 2015). Furthermore, Gamsakhurdia (2019) states that the transition of identity in different cultures is “expected to result in the breaking of the initial opposition between A (native identity) and non-A (host identity) using a constructive mixture of opposites and the creation of a new higher-level sign.” Gamsakhurdia (2019, 2022) also proposes the term “proculturation,” which refers to a dynamic process that evolves in “three-dimensional temporal space–past< >present< >future.” This process typically refers to the cultural and identity integration that occurs when individuals or groups from different cultural backgrounds communicate. In line with these perspectives, psychological well-being across different cultures can be comprehended as the ideal stage of cultural identity development.

In positive psychology, identity development involves overcoming conflicts that arise at different stages of life, influenced by societal factors and personal growth. The nature of identity is a person’s unconscious sense of self, both as an individual and a member of the greater community (Schaetti, 2015). The personal growth aspect of psychological well-being was derived from Erikson’s (1959) identity development theory, which emphasizes the importance of self-awareness and social roles in forming one’s identity. As Erikson (1959) states, “identity formation can be said to have a self-aspect and an ego aspect’.” Self-identity arises from experiences in which a feeling of temporary self-dissolution is effectively managed through renewed and increasingly authentic self-definition and social acknowledgment (Erikson, 1959). Identity development is closely related to psychological well-being, which involves feeling comfortable with one’s body, having a clear direction in life, and feeling confident about receiving recognition from significant others. However, an ideal sense of identity is neither achieved nor sustained. “Self-construction” and “self-discovery” are two ways to tackle identity development in the life span (Waterman, 1984, 2011). Self-construction refers to selecting valuable components from various options. Self-discovery is a journey of discovering the true self with better identity choices, which is “closely linked with eudaimonist philosophy (Waterman, 2011).” Waterman et al. (2010) propose the Questionnaire for Eudaimonic Well-Being (QEWB), which delves into personal growth, self-acceptance, autonomy, and purpose in life. The QEWB includes high internal consistency, with score distribution approximately normal. It was assessed among university students from diverse racial backgrounds at multiple locations in the United States, indicating that the instrument has broad application and is a reliable tool for assessing eudaimonic well-being in diverse contexts.

Furthermore, gaining insight into individual experiences leads to a sense of purpose, resilience, and fulfillment in identity development, ultimately contributing to a more balanced and fulfilling life. Eriksson and Frisén (2024) state that personal experiences positively or negatively affect psychological well-being. Their research showed that a positive narration of personal experiences positively impacted personal growth and improved life satisfaction. By contrast, negative narration causes more psychological distress, which has a negative effect on identity development (Eriksson, 148 McLean and Frisén, 2020). Other studies have indicated that personal narratives are an effective method for exploring psychological well-being (Vanaken et al., 2022; Syed and McLean, 2022), identity development (Castelló et al., 2023), and cross-cultural issues (Sharif and Channa, 2022; Stetz, 2023). These studies show that personal experiences, stories, and how an individual addresses them are closely related to cultural identity and well-being because individuals use personal narratives to shape their identity while being influenced by their identity in the stories they choose to tell. Besides, narrative theming is effective in exploring identity development. According to Saldaña (2016), narrative analysis is particularly suitable for inquiries into identity development and psychological, social, and cultural meanings and values. It organizes and presents the meaning or subject of data units and is an appropriate approach for psychological attributes such as “beliefs, constructs, identity development, and emotional experiences” (Saldaña, 2016). Therefore, integrating personal experiences through narrative analysis theming offers a powerful tool for understanding the complex interplay between individual identity development and psychological well-being.

### 2.2 Psychological well-being and critical thinking

Psychological well-being is a diverse and multidimensional concept that has been researched and improved over the years. Ryff’s (1989) renowned work interpreted psychological well-being from a more eudaimonic perspective. The eudaimonic approach is rooted in Aristotle’s conception of happiness: Living well and doing well— living a virtuous life, pursuing noble actions, and enjoying appropriate happiness (Vittersø, 2016). The philosophical framework for exploring the complexities of “Happiness” might offer insights into understanding the eudaimonic approach to psychological well-being. Haybron (2000) stated that the comprehension of happiness cannot rely solely on conceptual analysis but must start from a basis that satisfies both theoretical and practical needs. He presents three concepts of happiness, providing a philosophical framework for further clarifying eudaimonic well-being: Psychological Happiness (subjective well-being and emotional satisfaction), Prudential Happiness (living a virtuous life), and Perfectionist Happiness (a moral and admirable life that is good in all respects). Consistent with Haybron’s (2000) perspective, true well-being is closely related to Perfectionist Happiness. Haybron (2016) attempts to establish a logical foundation for incorporating various ultimate goods and provides three norms that should be embodied in well-being: admirability (perfection), actualization (capacity fulfillment), and success (goal fulfillment). Nonetheless, he claims that the Aristotelian theme of well-being struggles to balance the concepts of actualization and admirability in individuals’ goal fulfillment. Moreover, Ford et al. (2015) showed that, to some extent, the motivation to pursue happiness can have negative consequences for well-being. This raises the question of how virtuous activity fits into well-being, while still prioritizing the idea of exercising our capacities.

Positive psychology offers a more holistic perspective on the factors contributing to an individual’s well-being. It emphasizes positive experiences, personal characteristics, and supportive institutions that promote human flourishing and optimal performance. The eudaimonic literature is extensive, but three critical approaches are frequently referenced in the psychological field: Psychological Well-Being (Ryff, 1989; Ryff and Singer, 2008), Personal Expressiveness (Waterman, 1984, 1993), and Self-Determination Theory (Ryan and Deci, 2001). The eudaimonic approach in psychology suggests that true well-being, which is much more than happiness, lies in the “positive psychological functioning” (Ryff, 1989), the “feeling of personal expressiveness” (Waterman, 1993), and the “actualization of human potentials” (Ryan and Deci, 2001). Ryff (2014) proposed revised dimensions of psychological well-being, including autonomy, environmental mastery, personal growth, positive relationships with others, purpose in life, and self-acceptance. Furthermore, Ryff (2022, 2023a) advocates for linking the eudaimonic well-being research with challenges in life and offers further interpretations of the “embedding of the negative within the positive.” Ryff (2023a, 2022, 2014) addressed the significance of certain individual qualities in dealing with real-life complexities, such as compassion, resilience, or adaptation. In line with that, one should engage in a self-fulfillment process involving effortful and goal-directed thinking to achieve true well-being. René Descartes’ philosophical statement, “Cogito, ergo sum” (which translates to, “I think, therefore I am”), highlights the importance of thinking for one’s existence. Kahneman (2011) proposes the idea of two types of thinking: System 1 thinking (“operates automatically and quickly, with little or no effort and no sense of voluntary control”) and System 2 thinking (“allocates attention to the effortful mental activities that demand it, including complex computations”). From this standpoint, hedonic well-being encompasses System 1 thinking, which is characterized by automaticity and effortlessness, while eudaimonic well-being embodies System 2 thinking, which is more mindful and effortful.

Critical thinking is System 2 thinking (Halpern 2014). It is a cognitive process that enables one to make reasoned judgments and decisions through analyzing, evaluating, and interpreting information. According to Halpern (2014), critical thinking “is used to describe thinking that is purposeful, reasoned, and goal-directed—the kind of thinking involved in solving problems, formulating inferences, calculating likelihoods, and making decisions when the thinker is using skills that are thoughtful and effective for the particular context and type of thinking task.” Barnett (2021) suggests that critical thinking can be comprehended in mind, behavior, and being. Just like those interrelatedly connected staircases in the painting of M.C. Esher, these three parts are “interfused” and work together for new possibilities for decisions, new opportunities for beings, and new options for applying critical thinking skills (Barnett, 2021). In light of that, attaining the ideal self is only possible by intentionally practicing critical thinking. Thus, critical thinking could be comprehended as a more extensive concept like “criticality” or “critical being” (Barnett, 2021). Cultural identity development is a continuous self-exploration and maturation process requiring critical thinking. Critical thinking enables individuals to analyze and evaluate diverse viewpoints within a multicultural context, fostering inclusivity and respect. It is essential for constructing an authentic, inclusive, and informed cultural identity. According to Côté (2015), the understanding of critical thinking applied to identity studies reveals that identity development is often misunderstood and confused with identity maintenance. If identity development is a process of “purpose practice,” as Moran (2009, 2020) suggested, cognitive growth should be considered. Cultural identity development requires intellectual breakthroughs by applying critical thinking, which entails challenging stereotypes, questioning societal norms, and actively seeking diverse perspectives.

Critical thinking and beliefs are intertwined intricately with deductive reasoning. Although a personal conviction may affect deductive reasoning, for it to be deemed accurate, it must conform to the principles of reasoning (Halpern, 2014). Dwyer (2017) also argued that “when deciding what to believe, we must avoid relying solely on justifications to accept our beliefs and refrain from merely seeking to affirm them, which results in a confirmation bias. This necessitates not only the determination of our own beliefs but also a critical examination of those beliefs.” In the intercultural theories, Byram (2012, 2021) concept of critical cultural awareness in second language acquisition emphasizes developing critical skills and reflexivity, which means critically evaluating our and others’ beliefs, values, and behaviors to comprehend cultural differences. Therefore, the connections between critical thinking and beliefs are supposed to impact the perception of cultures. Developed by Stupple et al. (2017) to investigate attitudes and beliefs regarding critical thinking, CriTT demonstrates strong psychometric properties and validity, encompassing three psychological elements of critical thinking: confidence in critical thinking (CCT), Valuing Critical Thinking (VCT), and misconceptions of critical thinking (MCT) (Stupple et al., 2017). CCT and VCT serve as bridges, allowing effective communication and understanding across different cultural contexts, and MCT is an obstacle to becoming a critical thinker. These factors are closely related to the theoretical and practical aspects of critical thinking and can predict academic performance. Although the CriTT was developed within the context of psychology courses, its content and applications are not necessarily limited to that discipline. Stupple et al. (2017) removes all items directly referencing psychology, indicating that the CriTT tool can be applicable across various fields of study.

### 2.3 Psychological well-being and cultural intelligence

In today’s cultural context, enhancing psychological well-being requires individuals to overcome many linguistic, cultural, and identity challenges. This requires individuals to navigate through various obstacles, such as language barriers, cultural differences, and the struggle to establish a sense of identity. Oxford (2018) proposed the EMPATHICS model of language learner well-being to address this issue. EMPATHICS modal comprises (E)motions (including pleasant and painful emotions) and empathy, (M)eaning and motivation, (P)erseverance (1 - hope, 2 - optimism, 3 - resilience), (A)gency and autonomy, (T)ime, (H)ardiness and habits of mind, (I)ntelligence, identity, investment, imagination, (C)haracter strengths, and (S)elf-components (self-efficacy, self-concept, self-esteem, self-regulation) (Oxford, 2018). This model illustrates how a language learner achieves well-being in an intercultural context. The intelligence components represent emotional intelligence, a personal characteristic crucial for enhancing overall well-being (Harzer, 2016). However, Ang et al. (2011) proposed that the emotional quotient (EQ) is contingent on culture, and cultural intelligence (CQ) is not limited to a particular culture; it encompasses a broad range of skills applicable to diverse cultural contexts. Asser and Langbein-Park (2015) also stated that the CQ combines the intelligence quotient (IQ), physical quotient (PQ), and EQ. Among these three, CQ shares much in common with EQ. Therefore, developing cultural intelligence is essential for individuals to successfully navigate the complexities of various cultures and improve their psychological well-being.

Cultural intelligence (CQ) is defined as “an individual’s capability to function and manage effectively in culturally diverse settings (Ang et al., 2007).” Sternberg (1997) proposes that CQ is the ability to “grasp., reason, and behave” appropriately and sensibly in contexts of cultural differences. Sternberg et al. (2022) further clarify that the nature of cultural intelligence is “not something one can memorize, such as a vocabulary list or a set of facts. Rather, it is something that one deploys according to the intricacies of a situation and in light of the task and the persons involved.” CQ consists of four factors: metacognitive, cognitive, motivational, and behavioral CQ. Metacognitive CQ refers to one’s ability to learn and comprehend other cultures. Cognitive CQ is the knowledge of rules, traditions, and customs in various cultural contexts. Motivational CQ is the capacity to concentrate attention and energy toward acquiring and functioning cultural knowledge in intercultural environments. Behavioral CQ is the competence to perform verbal and nonverbal actions appropriately in intercultural contexts (Ang et al., 2007). Becoming culturally intelligent means developing cultural adaptability to interact with cultures, people, and intercultural contexts (Asser and Langbein-Park, 2015). Regarding intercultural context, Asser and Langbein-Park (2015) states that the individual’s CQ represents the improvement of “intercultural sensitivity, intercultural communication skills, the ability to build commitment and manage uncertainty.”

Although CQ has a short history, its importance has been revealed in many disciplines. Fang et al.’s (2018) review of 142 articles on CQ reveals that it is an extensively researched concept that has increasingly caught the attention of psychology, education, and business. Over the past two decades, CQ research has primarily focused on two aspects: the application of CQ in different contexts and the connection between individual attributes and CQ. First, CQ has been studied in various educational and business contexts (Suthatorn and Charoensukmongkol, 2018; Wang, 2021; McKay et al., 2022; Daniel, 2022; Alexandra, 2022; Iskhakova et al., 2022; Dolce et al., 2023; Wolff et al., 2023; Skaria and Montayre, 2023). Second, researchers have investigated the relationship between CQ and a wide range of individual traits, such as anxiety level (Suthatorn and Charoensukmongkol, 2018; Bernardo and Presbitero, 2018), cognitive flexibility (Bernardo and Presbitero, 2018), intercultural competence (Li, 2020), foreign language skills (Presbitero, 2020), international experience (Ott and Iskhakova, 2019; McKay et al., 2022; Pidduck et al., 2022), and personality (Chédru and Ostapchuk, 2023). Moreover, the interconnection between CQ and EQ is also a frequently studied topic, which has been explored in various circumstances (Lin et al., 2012; Darvishmotevali et al., 2018; Lam et al., 2021; Cheung et al., 2022; Musarra et al., 2022; Davaei et al., 2022). Cultural intelligence should be considered an important element to enhance the psychological well-being between cultures. Pidduck et al.’s (2022) model presents the relationship between cross-cultural experience, cultural intelligence, and multicultural identity, but more evidence is needed for how CQ interacts with other components in cultural identity construction.

Various measurements are developed to assess cultural intelligence. Ang et al.’s (2007) Cultural Intelligence Scale (CQS) is the most popular measurement of cultural intelligence. The CQS contains 20 items and assesses four core dimensions of cultural intelligence: Metacognitive CQ, Cognitive CQ, Motivational CQ, and Behavioral CQ. Because CQS has been validated in two different cultural systems, the United States and Singapore, it has cross-validation and stability. However, Thomas et al. (2015) argues that the CQS has two problematic features. First, the CQS is incapable of addressing the relationship between each dimension. Second, the overall CQS construct needs to reveal how these four dimensions aggregate and interact. Compared with the CQS, the short-form measure of cultural intelligence (SFCQ) developed by Thomas et al. (2015) has fewer dimensions and items. This 10-item scale explores three significant cultural intelligence elements: metacognition, knowledge, and skills. Cultural metacognition is the central element of CQ and involves the mental processes of awareness, conscious analysis, and evaluation of the solution to a problem. Cultural knowledge refers to specific and processed knowledge related to recognizing cultural differences, comprehending intercultural encounters, and adapting to various cultures. Cultural skills are a series of abilities associated with acquiring knowledge through intercultural communication, connecting with people from other cultures, and adapting to various cultural situations (Thomas et al., 2015). SFCQ is related to emotional intelligence (EQ) and personality traits but is distinct from them. It positively correlates with indicators of multicultural experience and the capacity to predict sociocultural adaptation, work performance in multicultural environments, and accurate interaction in cross-cultural interactions.

## 3 Methods

### 3.1 Research design

This research has two aims. The first is to explore how critical thinking beliefs and cultural intelligence interact to influence individuals’ eudaimonic well-being. The second focuses on understanding how critical thinking, cultural intelligence, and eudaimonic well-being contribute to a sense of purpose and fulfillment in cultural and identity-related challenges. Two separate phases of the research were conducted at various times, and Study 1 was conducted one year before Study 2.

In the quantitative analysis in Studies 1 and 2, four questionnaires are applied: the Critical Thinking Toolkit (CriTT, Stupple et al., 2017); Cultural Intelligence Scale (CQS, Ang et al., 2007); Short Form of Cultural Intelligence (SFCQ, Thomas et al., 2015); and Questionnaire for Eudaimonic Well-Being (QEWB; Waterman et al., 2010). The CriTT was adopted in the quantitative part of both Studies 1 and 2. Both the CQS and SFCQ were applied in this research to investigate how every dimension of cultural intelligence interacted with critical thinking belief. QEWB is applied to investigate the relationship between Eudaimonic Well-Being, critical thinking belief, and cultural intelligence. Study 1 involved the quantitative analysis using Questionnaire 1, which contained 27 CriTT items and 10 SFCQ items. In Study 2, Questionnaire 2 consisted of 68 items, including 27 from the CriTT, 20 from the CQS, and 21 from the QEWB. All questionnaires were rated on a 5-point Likert scale. The CriTT was administered to art students, providing evidence for its application across different disciplines and cultural contexts. The original QEWB shows a near-normal distribution of scores, and this study tries to investigate further how critical thinking beliefs and cultural intelligence affect the measurement of well-being. Correlation analyses using SPSS were conducted between the CQS, SFCQ, CriTT, and QEWB to investigate the connections between critical thinking, cultural intelligence, and eudaimonic well-being.

The second section of Study 2 involved a qualitative analysis of participants’ narratives on well-being using NVivo 20.0. The narrative data were collected from a writing assignment that required students to reflect on their attitudes and thoughts about well-being. Based on Ryff’s (1989, 2014) model, the assignment includes some questions to guide students in constructing their sense of well-being: 1. What is a happy life in your mind? 2. How do you think happiness in different cultures? 3. What is the most significant accomplishment in your life? 4. How can you contribute to the well-being of humans? Students were encouraged to answer open-ended questions freely, which were then included in the assignment, ensuring that data collection captured rich, objective information for qualitative analysis.

### 3.2 Samples

This research included 821 participants who were freshmen in an arts college. Informed consent was obtained from participants before the study, and the ethical committee approved the study protocol. In Study 1, to explore the relationship between critical thinking beliefs and cultural intelligence, Questionnaire 1 was administered to 510 first-year students and analyzed using SPSS 20.0. Based on the findings of Study 1, Questionnaire 2 was administered to 311 first-year students to validate the results of Study 1. In the qualitative part of Study 2, participants were selected from among those who received the highest score on Questionnaire 2.

### 3.3 Research tools and data collection

#### 3.3.1 Reliability and validation of Questionnaire 1

Questionnaire 1 comprises the CriTT and SFCQ, with 37 items divided into two parts. The first part has 27 items from the CriTT, among which 17 questions assess the level of Confidence in Critical Thinking (CCT), 6 for Valuing Critical Thinking (VCT), and 4 for Misconceptions of Critical Thinking (MCT, reverse scoring). The second part has 10 items from the SFCQ. Questions 1–2 concern cultural knowledge, 3–7 concern cultural skills, and 8–10 concern cultural metacognition. Cronbach’s Alpha was used to evaluate the internal consistency of Questionnaire 1. Table 1 shows that Cronbach’s Alpha for the CriTT was 0.887, for the SFQS was 0.931, and for the whole questionnaire was 0.934. For standardized items, the CriTT was 0.905, the SFQS was 0.931, and the whole questionnaire was 0.943. Additionally, the Cronbach’s alpha values of the original CriTT (Stupple et al., 2017) were 0.92 for CCT, 0.79 for VCT, and 0.60 for MCT, and the version in Questionnaire 1 shows higher reliability (CCT: 0.949, VCT: 0.804, MCT: 0.686) than the original one. Thomas et al. (2015) reported that the original SFCQ had a Cronbach’s *α* of 0.88, and the reliability of the SFCQ was found to be 0.931 in Questionnaire 1. These results reflect that Questionnaire 1 was highly reliable.

**Table 1:** The validation of Questionnaire 1.

	Cronbach’s alpha	Cronbach’s alpha based on standardized items	N of items
CriTT	0.887	0.905	27
CCT	0.949	0.951	17
VCT	0.804	0.817	6
MCT	0.686	0.687	4
SFCQ	0.931	0.931	10
Questionnaire 1	0.934	0.943	37

#### 3.3.2 Reliability and validation of Questionnaire 2

Questionnaire 2 comprises the CriTT, CQS, and QEWB, with 68 items divided into three parts. The first part has 27 items from the CriTT. The second part comprises 20 items from the CQS. Questions 1–4 concern Metacognitive CQ (MC), 5–10 concern Cognitive CQ (COG), 11–15 concern Motivational CQ (MOT), and 16–20 concern Behavioral CQ (BEH). The third part has 21 items from QEWB. As seen in Table 2, Cronbach’s Alpha for the CriTT was 0.920, for the CQS was 0.971, for the QEWB was 0.928, and for the whole questionnaire was 0.976. For standardized items, the CriTT was 0.929, the CQS was 0.972, the QEWB was 0.935, and the whole questionnaire was 0.978. Moreover, compared to Cronbach’s alpha values of the original CriTT (CCT: 0.92, VCT: 0.79, MCT: 0.60), the current CriTT dimensions exhibit higher reliability (CCT:0.968, VCT: 0.869, MCT:0.737). The current CQS demonstrates higher reliability across all dimensions (MC: 0.914, COG: 0.951, MOT: 0.928, BEH: 0.953) compared to the original one (MC: 0.71–0.77, COG: 0.8–0.88, MOT: 0.77–0.79, BEH: 0.82–0.84, Ang et al., 2007). Waterman et al. (2010) reported that the original QEWB had a Cronbach’s alpha of 0.86, while the QEWB in Questionnaire 2 shows a higher Cronbach’s alpha of 0.928. Table 2 indicates that Questionnaire 2 has improved reliability over the original CriTT, CQS, and QEWB.

**Table 2:** The validation of Questionnaire 2.

	Cronbach’s alpha	Cronbach’s alpha based on standardized items	*N* of items
CriTT	0.920	0.929	27
CCT	0.968	0.968	17
VCT	0.869	0.878	6
MCT	0.737	0.737	4
CQS	0.971	0.972	20
MC	0.912	0.914	4
COG	0.951	0.951	6
MOT	0.928	0.928	5
BEH	0.953	0.953	5
QEWB Questionnaire 2	0.928 0.976	0.935 0.978	21 68

#### 3.3.3 The selection of participants for qualitative analysis in Study 2

**Figure 1:**
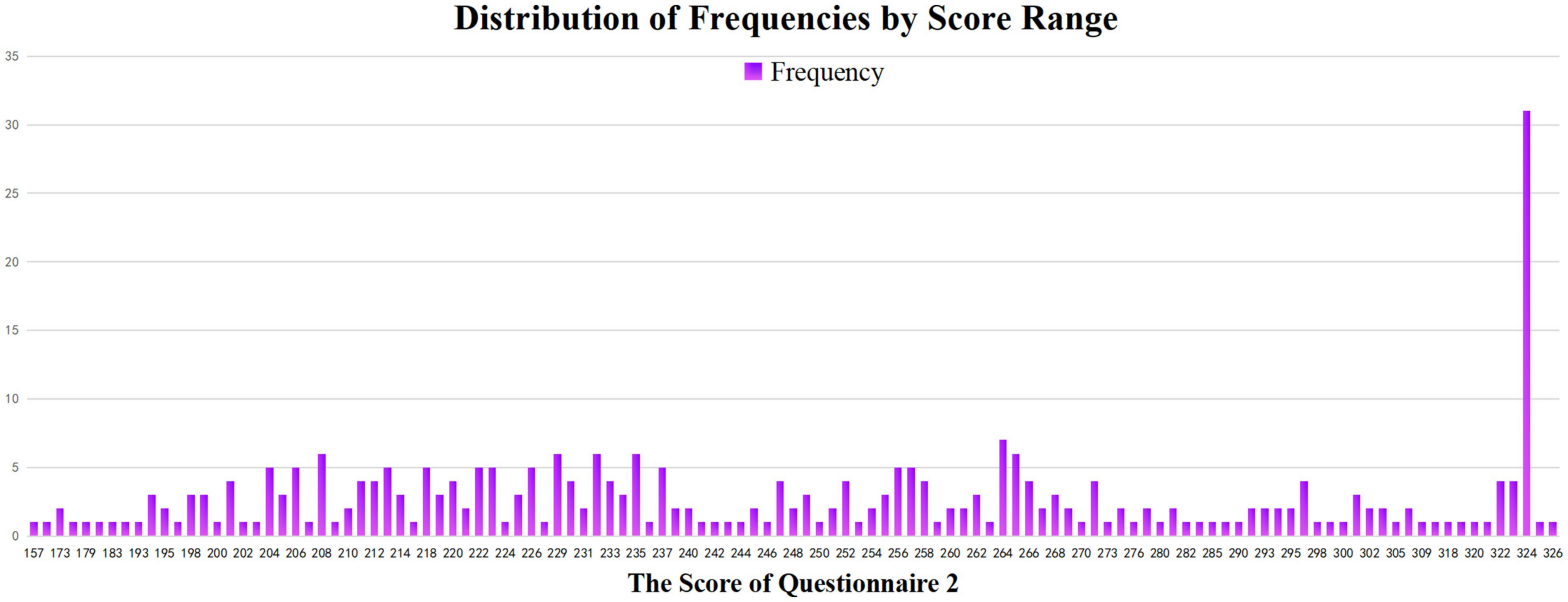
Distribution of frequencies by score range.

Table 3 shows that the maximum Questionnaire 2 score was 326, and the minimum was 157. Figure 1 shows that approximately 35 participants are clustered in the score range between 322 and 326. Thirty assignments from the participants with the highest score in Questionnaire 2 were randomly selected to analyze in NVivo 20.0.

**Table 3:** Descriptive statistics of the variables.

	*N*	Minimum	Maximum	Mean	Std. deviation
The total score of Questionnaire 2	311	157	326	252.408360	41.7896861
Valid N (listwise)	311				

#### 3.3.4 Narrative analysis

The narrative analysis of qualitative research in Study 2 includes open, axial, and selective coding to refine the core themes. The narrative coding strategies referenced Charmaz’s (2014) identity analytic categories in grounded theory, ensuring the findings were grounded in the raw data. According to Squires (2023), six-steps of thematic analysis enhance the trustworthiness of qualitative data: “1. familiarizing oneself with the data, 2. generating initial codes, 3. searching for themes, 4. reviewing themes, 5. defining and naming themes, and 6. reporting findings.” In Study 2, after the raw data collection, the open-coding process began with an in-depth engagement with the data, during which key terms and phrases were identified as initial codes. The initial codes act as the basis for further analysis, and the complexity and richness of participants’ experiences are organized and concluded in this phase. Axial coding was then applied to connect these concepts and develop the important categories and subcategories, which were refined and integrated into the core themes for further review, refining, and modification. Finally, these core themes were defined and named, ultimately demonstrated in the research findings.

## 4 Results

### 4.1 The findings of Study 1

#### 4.1.1 Relationship between critical thinking belief (CTB) and cultural intelligence (CQ)

Pearson’s correlation (SPSS 20.0) was applied to analyze the relationship between critical thinking beliefs (CTB) and the CQ dimensions (see Table 4). The relationships among Confidence in Critical Thinking (CCT), Valuing Critical Thinking (VCT), Misconceptions about critical thinking (MCT), Cultural Knowledge (CK), Cultural Skills (CS), and Cultural Metacognition (CM) were analyzed. As shown in Table 4, the correlation between these dimensions ranged from −0.360 to 0.842, and each correlation coefficient was statistically significant (*p* < 0.01). Among them, the total score of CTB and CQ had a strong positive correlation (*r* = 0.761, *p* < 0.01); For the three dimensions of CTB, CCT was positively related to CK (*r* = 0.592, *p* < 0.01), CS (*r* = 0.717, *p* < 0.01), CM (*r* = 0.764, *p* < 0.01), and total score of CQ (*r* = 0.770, *p* < 0.01); VCT was also positively related to CK (*r* = 0.595, *p* < 0.01), CS (*r* = 0.615, *p* < 0.01), CM (*r* = 0.600, *p* < 0.01), and total score of CQ (*r* = 0.660, *p* < 0.01). However, MCT were negatively related to CK (*r* = −0.360, *p* < 0.01), CS (*r* = −0.515, *p* < 0.01), and CM (*r* = −0.501, *p* < 0.01), and total score of CQ (*r* = −0.522, *p* < 0.01).

**Table 4:** Pearson correlation for all the study variables (*n* = 510).

	CCT	VCT	MCT	CTB	CK	CS	CM
CCT	–						
VCT	0.759**	–					
MCT	−0.645**	−0.558**	–				
CTB	0.968**	0.844**	−0.499**	–			
CK	0.592**	0.595**	−0.360**	0.622**	–		
CS	0.717**	0.615**	−0.515**	0.702**	0.692**	–	
CM	0.764**	0.600**	−0.501**	0.743**	0.620**	0.842**	–
CQ	0.770**	0.660**	−0.522**	0.761**	0.793**	0.968**	0.924**

**p < 0.01 (2-tailed).

Moreover, strong positive correlations are also founded between CCT and the total score of CTB (*r* = 0.968, *p* < 0.01), between CK and the total score of CQ (*r* = 0.968, *p* < 0.01), and between CM and total score of CQ (*r* = 0.924, *p* < 0.01). MCT was not only negatively related to every dimension of SFCQ but also negatively related to CCT (*r* = −0.645, *p* < 0.01), VCT (*r* = −0.558, *p* < 0.01), and the total score of CTB (*r* = −0.499, *p* < 0.01). Funder and Ozer (2019) states that if r = 0.4 or above, the effect size in psychological research can be very substantial, indicating that this study’s result could provide explanatory insights into the relationship between critical thinking and CQ. The results of Study 1 indicate significant correlations between critical thinking beliefs and cultural intelligence. The result of this part suggests that being confident when applying critical thinking or attaching importance to critical thinking will significantly affect acquiring cultural knowledge, operating cultural skills, and performing metacognition. Additionally, the misunderstanding of critical thinking will considerably affect the application of critical thinking and cultural intelligence.

### 4.2 The findings of Study 2

#### 4.2.1 Results of quantitative analysis in Study 2

##### 4.2.1.1 Relationship between critical thinking belief (CTB), cultural intelligence (CQ), and eudaimonic well-being (EWB)

Table 5 reveals a significant positive correlation between CTB and CQ (*r* = 0.872, *p* < 0.01). This result aligns with that reported in Table 4. Both tables demonstrate a strong relationship between critical thinking belief and cultural intelligence, regardless of measurement method (SFCQ or CQS). Additionally, EWB was positively related to CQ (*r* = 0.833, *p* < 0.01) and CTB (*r* = 0.728, *p* < 0.01). This result indicates that individuals with strong critical thinking beliefs and high cultural intelligence are likely to have higher levels of eudaimonic well-being.

**Table 5:** Pearson correlation for critical thinking belief (CTB), cultural intelligence (CQ) and eudaimonic well-being (EWB).

	CTB	CQ	EWB
CTB	–	0.872**	0.728**
CQ	0.872**	–	0.833**
EWB	0.728**	0.833**	–

**p < 0.01 (2-tailed).

##### 4.2.1.2 Relationships between eudaimonic well-being (EWB) and each dimension of critical thinking belief and cultural intelligence

Pearson’s correlation (SPSS 20.0) was still used to examine the connections between eudaimonic well-being and each facet of critical thinking belief and cultural intelligence. Table 6 displays the correlation between eudaimonic well-being (EWB), Confidence in Critical Thinking (CCT), Valuing Critical Thinking (VCT), Misconceptions about critical thinking (MCT), Metacognitive CQ (MC), Cognitive CQ (COG), Motivational CQ (MOT), and Behavioral CQ (BEH). The research findings varied from −0.523 to 0.844; each correlation coefficient was statistically significant (*p* < 0.01). As shown in Table 6, EWB is positively related to all other dimensions. The results demonstrate that EWB has a strong positive correlation with CCT (*r* = 0.766, *p* < 0.01), VCT (*r* = 0.698, *p* < 0.01), MC (*r* = 0.725, *p* < 0.01), COG (*r* = 0.763, *p* < 0.01), MOT (*r* = 0.739, *p* < 0.01), and BEH (*r* = 0.762, *p* < 0.01). Moreover, EWB strongly correlates negatively with MCT (*r* = −0.711, *p* < 0.01).

**Table 6:** Pearson correlation for eudaimonic well-being (EWB) and each dimension of critical thinking belief (CTB) and cultural intelligence (CQ) (*n* = 311).

	EWB	CCT	VCT	MCT	MC	COG	MOT
EWB	–	–	–	–	–	–	–
CCT	0.766**	–	–	–	–	–	–
VCT	0.698**	0.844**	–	–	–	–	–
MCT	−0.711**	−0.692**	−0.678**	–	–	–	–
MC	0.725**	0.846**	0.744**	−0.599**	–	–	–
COG	0.763**	0.786**	0.677**	−0.635**	0.726**	–	–
MOT	0.739**	0.807**	0.713**	−0.547**	0.732**	0.751**	–
BEH	0.762**	0.766**	0.701**	−0.523**	0.721**	0.718**	0.810**

**p < 0.01 (2-tailed).

Among the dimensions of CriTT, CCT was positively related to VCT (*r* = 0.844, *p* < 0.01). MCT were negatively related to CCT (*r* = −0.692, *p* < 0.01) and VCT (*r* = −0.678, *p* < 0.01). Among the dimensions of CQS, MC is positively related to COG (*r* = 0.726, *p* < 0.01), MOT (*r* = 0.732, *p* < 0.01), and BEH (*r* = 0.721, *p* < 0.01). COG is positively related to MOT (*r* = 0.751, *p* < 0.01) and BEH (*r* = 0.718, *p* < 0.01). MOT is positively related to BEH (*r* = 0.810, *p* < 0.01). Table 5 demonstrates the strong correlation between critical thinking beliefs and cultural intelligence. In Table 6, CCT is positively related to MC (*r* = 0.846, *p* < 0.01), COG (*r* = 0.786, *p* < 0.01), MOT (*r* = 0.807, *p* < 0.01), and BEH (*r* = 0.766, *p* < 0.01). VCT is also positively related with MC (*r* = 0.744, *p* < 0.01), COG (*r* = 0.677, *p* < 0.01), MOT (*r* = 0.713, *p* < 0.01), and BEH (*r* = 0.701, *p* < 0.01). Besides, strong negative correlations are also found between MCT and MC (*r* = −0.599, *p* < 0.01), COG (*r* = −0.635, *p* < 0.01), MOT (*r* = −0.547, *p* < 0.01), and BEH (*r* = −0.523, *p* < 0.01).

#### 4.2.2 Results of qualitative analysis in Study 2

**Figure 2:**
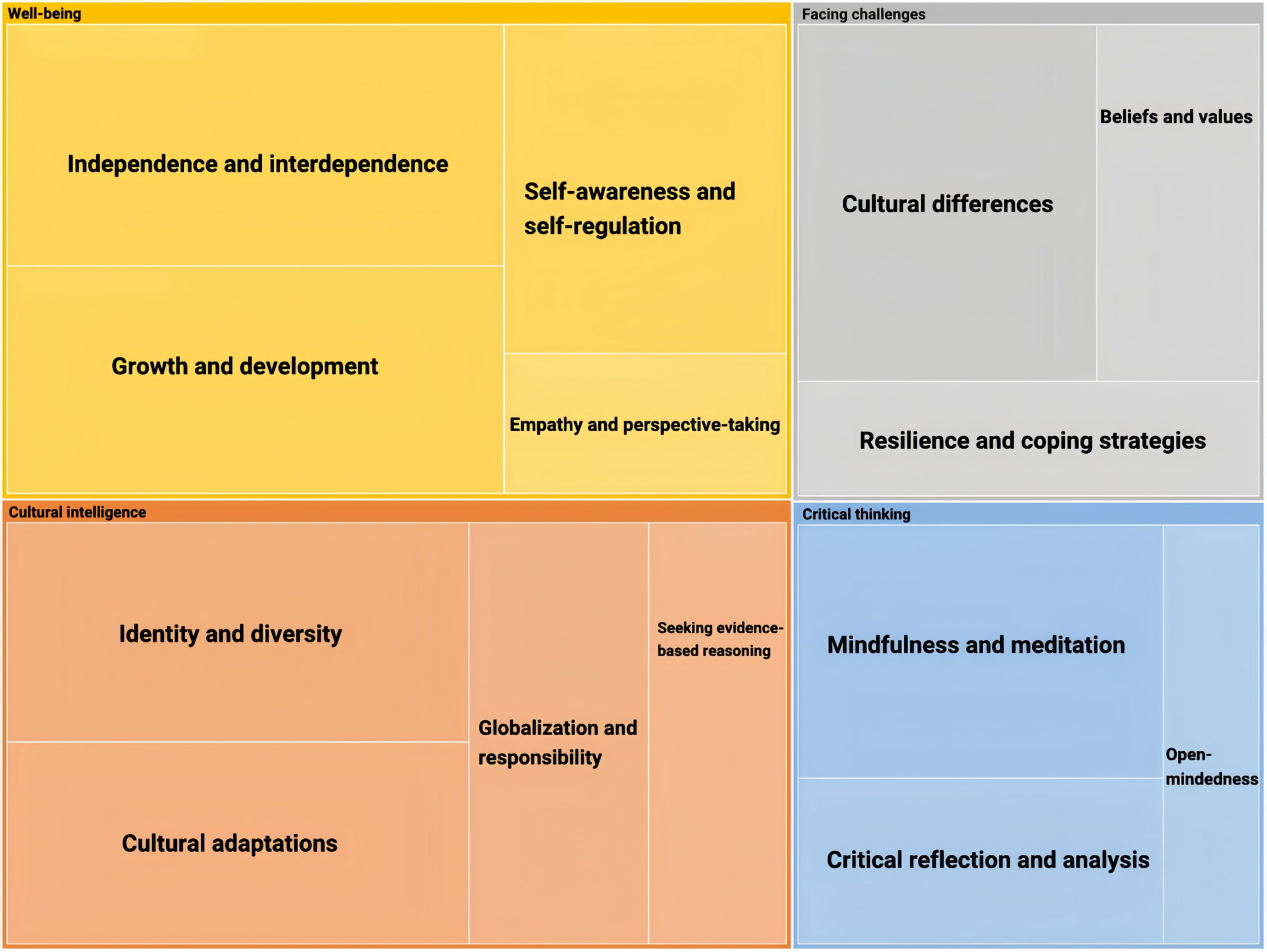
The themes of well-being, cultural intelligence, critical thinking, and facing challenges.

As Figure 2 demonstrates, the research findings indicate that the cultural well-being diagram comprises four different themes (Well-being, Cultural intelligence, Critical thinking, and Facing challenges), each representing a specific theme derived from the participants’ descriptive data.

**Figure 3:**
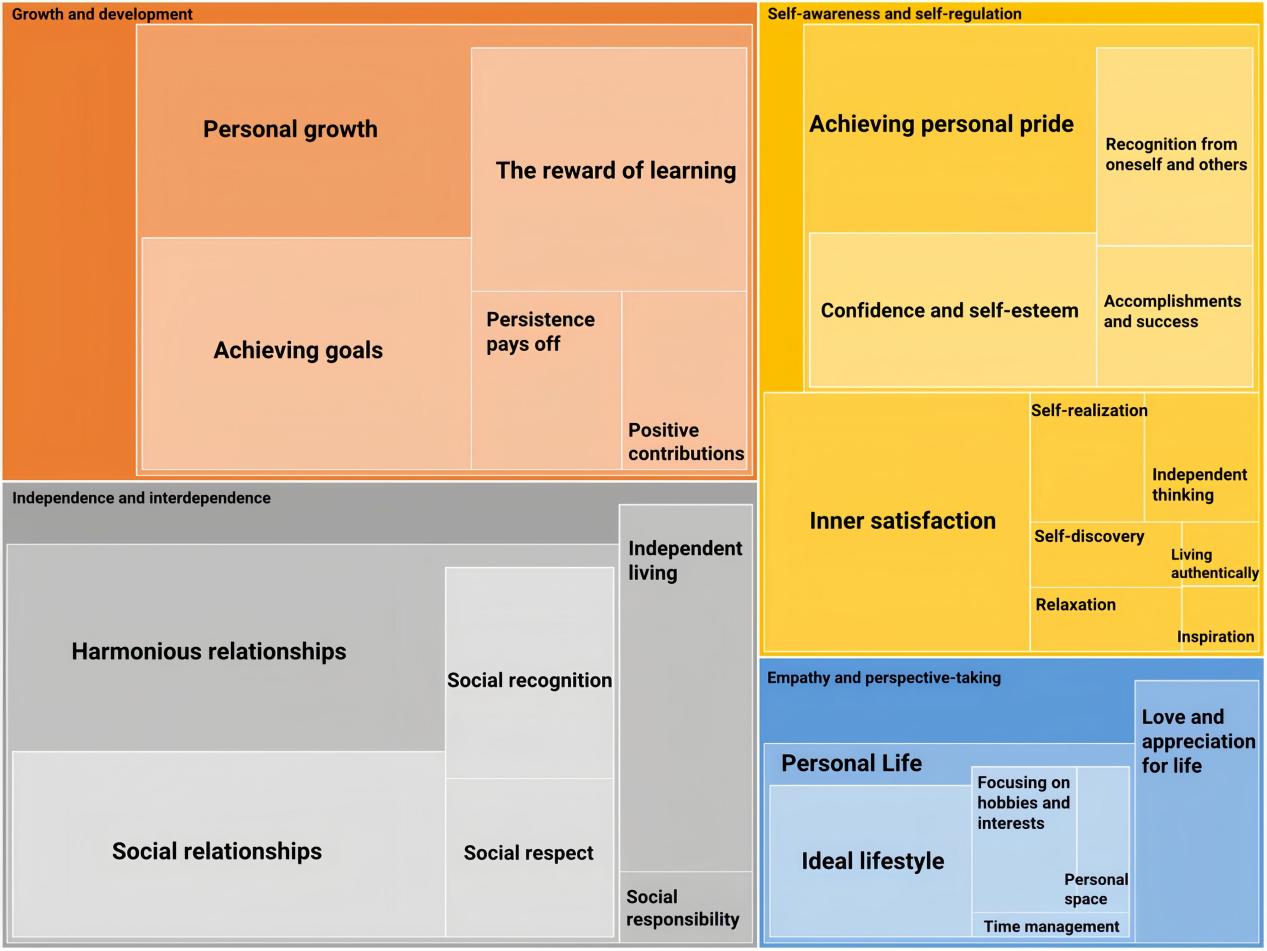
The theme of psychological well-being.

**Figure 4:**
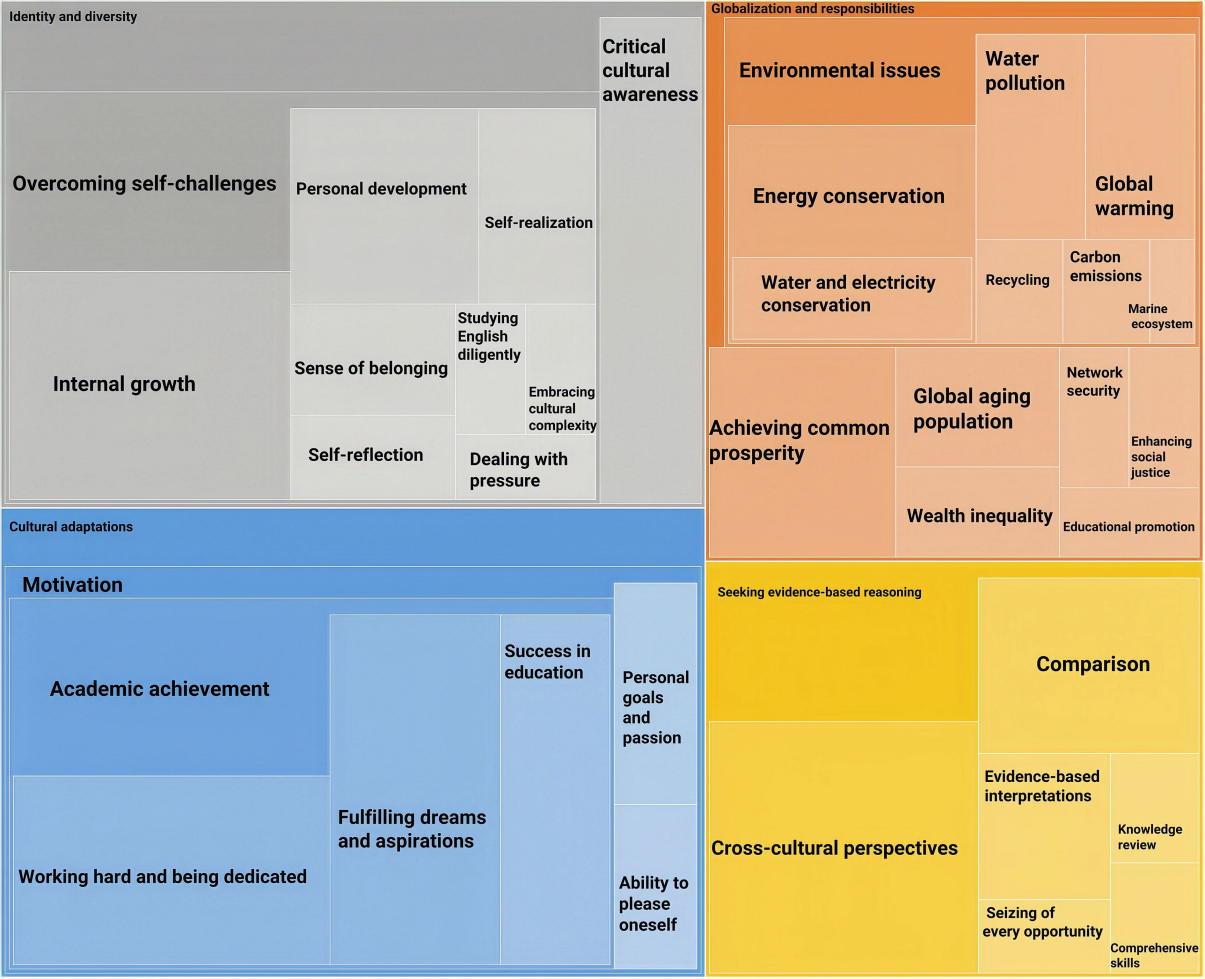
The theme of cultural intelligence.

**Figure 5:**
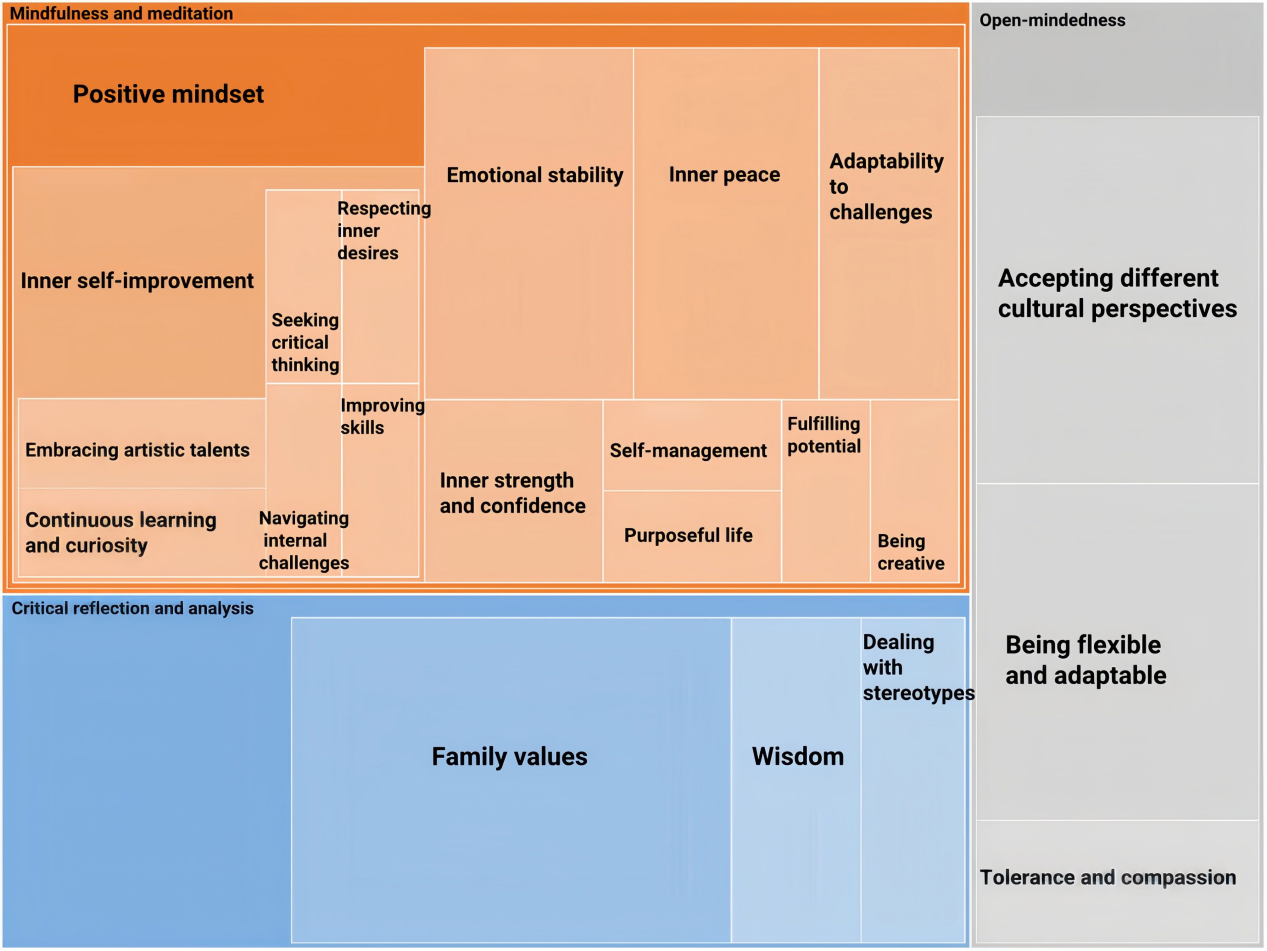
The theme of critical thinking.

The strategy to categorize identity should concern “selfhood, autonomy, legitimacy, and control.” (Charmaz, 2014) In detail, these concerns include “Embodiment and consciousness, Individual and collective action, Cooperation and conflict, Choice and constraint, Meanings and actions, Standpoints and differences, Ritual and ceremony, Positions and networks, Power and prestige, Structure and process, Opportunities and inequalities, Rights and resources, Moral life, moral action, and moral responsibility.”(Charmaz, 2014) In line with that, this research categorizes the themes of cultural identity in the scope of Well-being (Independence and interdependence, self-awareness and self-regulation, growth and development, empathy and perspective-taking), Cultural Intelligence (identity and diversity, cultural adaptations, globalization, and responsibility, seeking evidence and reasoning), Critical thinking (mindfulness and meditation, critical reflection and analysis, open-mindedness), and Facing challenges (cultural differences, beliefs and values, resilience, and coping strategies). These themes are demonstrated explicitly in Figures 2–5 and the samples of participants’ narrations. For research ethics, the participants are represented by S1, S2, S3,…., S30.

##### 4.2.2.1 The theme of psychological well-being

Figure 3 shows that the well-being theme contains four categories: Growth and development, Independence and interdependence, Empathy and perspective-taking, and Self-awareness and self-regulation. According to Ryff (2023a), research on eudaimonia should adopt a broader perspective, focusing on societal and community issues. These categories in Figure 3 are necessary elements of well-being (Ryff, 2014; Haybron, 2016). These elements contribute to individual well-being and the greater good, as individuals can enhance their personal development through altruism, community involvement, and helping those in need.

The part of Growth and development represents personal growth in coping with cultural difficulties. It is the process of expanding and improving oneself and advancing one’s skills and abilities. Figure 3 demonstrates that personal growth can be further generated in the four approaches: The reward of learning, Achieving goals, Persistence Pays Off, and Positive contributions. The reward of learning demonstrates that individual development can be achieved by continuous learning and obtaining new knowledge and skills.

*S17: To get the life you want in the future, you should always stay true to yourself and have new ideas. Time management is important, for it is the key to learning new things. Only in this way can one stay competitive and overcome the cultural boundaries of design inspiration.*

Achieving Goals is a way to foster personal development, experience a sense of accomplishment, and appreciate an individual’s potential. Persistence Pays Off emphasizes the importance of perseverance and resilience when confronted with obstacles and disappointments. Positive contributions involve actions that benefit both others and the global community. These two elements involve personal and societal approaches necessary for individual growth and a meaningful life.

*S10: My future life should be fulfilling and full of hope. I should be able to do what I like, have creativity and initiative, and have ample time for artistic creation. Achieving this dream will give my future more hope, increase my happiness, and make me feel fulfilled. Therefore, I need to work hard to achieve my vision of a dream life for the future.*

Independence and interdependence are other significant factors that influence well-being, which include three parts: Harmonious relationships, Independent living, and Social responsibility and contributions. Harmonious relationships encompass Social recognition, Social relationships, and Social respect, which are essential for maintaining positive emotional well-being. Social recognition involves the feelings acknowledged by others. Social relationships represent the interpersonal connections we establish with others, such as family, friends, and classmates. Social respect involves kindness, empathy, and understanding acquired through positive interactions with others. Harmonious relationships illustrate how participants develop self-esteem, confidence, and a sense of belonging. Independent living is another attribute that significantly affects one’s sense of autonomy and control over life. Social responsibility and contributions represent making a positive impact on society, providing participants with a chance to experience a profound sense of meaning, satisfaction, and affiliation with a higher entity. Independence and interdependence offer an angle for how individuals gain well-being by establishing connections between their ideal identity and that of others. Participants emphasize the capacity to live well and independently while contributing to society’s well-being.

Self-awareness and self-regulation illustrate the concept of the self in both Chinese and Western cultures. Self-awareness embodies the principle of “向内观己” (xiàng nèi guān jǐ) in Chinese philosophy, which can be comprehended as “introspection.” This refers to examining thoughts, feelings, and motivations to gain thoughtful insights into one’s identity. Self-regulation represents McAdams (2013) concept of “social actor” in personal identity establishment. McAdams (2013) proposes that from Freud’s (1961) view, self-regulation is a crucial “lifelong function, which is to observe the self and keep impulses in check.” It is essential for a “social actor” to learn to control impulses and think critically about every decision in identity development.

Figure 3 shows that Self-awareness and self-regulation encompass Achieving personal pride, Independent thinking, Inner satisfaction, Inspiration, Living authentically, Relaxation, Self-discovery, and Self-realization. Achieving personal pride comprises three factors: Accomplishments and success, Confidence and self-esteem, and Recognition from oneself and others. These elements demonstrate how participants cope with their personal goals, motivations, values, and future aspirations in their identity development process. They also combine the typical elements of Chinese cultural identity, such as Inner satisfaction, Relaxation, and Living authentically (Peng, 2023), which represent a comprehension of cultural identity deeply rooted in the Chinese philosophy of inner peace, harmony, and introspection. Independent thinking, inspiration, self-discovery, and self-realization demonstrate the conventional elements of Western cultural identity, such as independence, individualism, and inspiration-motivated thoughts (Peng, 2023).

*S4: Personal growth involves aspects such as self-awareness, emotional management, and independent thinking. I will regularly self-reflect, identify my strengths and weaknesses, and actively adjust my mindset to achieve this goal. Additionally, I will pay attention to my thinking patterns, cultivate the ability to think independently and strive to become a more mature and independent individual.*

In Figure 3, Empathy and perspective-taking demonstrate the opinion of taking good care of oneself, including Love and appreciation for life and Personal Life. According to Steger et al. (2008), there is a positive correlation between eudaimonic behavior and well-being, while the relationship between hedonic behavior and well-being is less consistent. Empathy and perspective-taking represent prioritizing self-care, which is crucial for holistic well-being and an effective way to maintain a healthy balance in life. Love and appreciation for life are critical self-care goals. Participants said that they could develop a positive mindset from small pleasures and expressing gratitude for daily life. Personal life in Figure 3 illustrates various aspects that contribute to well-being and consists of four elements: Focusing on hobbies and interests, Ideal lifestyle, Personal space, and Time management. Participants desired an ideal lifestyle that allowed them space and time to develop hobbies and interests, such as dance, playing instruments, or writing novels. They believed that having a positive perspective on the ideal lifestyle could be a guiding principle for creating a meaningful and fulfilling life. They also hoped to effectively manage their time for both professional and recreational pursuits. Many participants uphold the traditional Chinese principle of “修身养性” (xiū shēn yǎng xìng), which emphasizes personality development. This involves practicing self-discipline and fostering inner growth through ethical pursuits.

*S5: My future dream is to have a peaceful and harmonious family and a society filled with love and care. I would feel a sense of value and accomplishment if I could contribute positively to society, such as participating in volunteer activities or supporting charitable causes. Similarly, I would be delighted to provide my family with a warm and comfortable living environment, allowing them to feel my love and care. Of course, realizing this goal requires continuous learning and personal growth to improve my abilities and qualities. I believe achieving this dream has a significant impact on my well-being.*

##### 4.2.2.2 The theme of cultural intelligence

Figure 4 shows that the theme of cultural intelligence contains four categories: Identity and diversity, Cultural adaptations, Globalization and responsibilities, and Seeking evidence-based reasoning. These four illustrate what guides individuals to thrive in a diverse cultural context.

Identity and diversity involve complex interactions between original cultural identity and diverse cultural phenomena, beliefs, and traditions. This category consists of two elements: Critical cultural awareness and Overcoming self-challenges. Critical cultural awareness involves cultivating curiosity and openness and being willing to challenge and suspend preconceived beliefs about other cultures and one’s own (Holliday, 2011). This entails the recognition and appreciation of cultural differences in beliefs and traditions. Participants demonstrate their critical cultural awareness of well-being. They thought that there were some differences between well-being in the Chinese and Western cultures, but most concepts were similar, such as inner peace, self-satisfaction, and harmonious relationships with others.

*S15: Chinese people are influenced by Confucianism and Taoism, valuing concepts such as “benevolence” and “ritual.” They see the spiritual world as superior to material life and circumstances, viewing it as a spiritual experience beyond wealth and status. In the West, Democritus believed that “pleasure and discomfort” constituted the standard for what should and should not be done, with happiness being the joy of the spirit and peace of the soul. Well-being has a common definition in Chinese and Western cultures.*

Figure 4 illustrates that Overcoming self-challenges includes Internal growth, Personal development, Self-realization, Self-reflection, A sense of belonging, Dealing with pressure, Embracing cultural complexity, and Studying English diligently. These elements demonstrate how to confront biases, prejudices, and stereotypes toward cultural differences. By dealing with the pressure of cultural complexity and language problems, the participants revealed that internal growth, personal development, self-realization, self-reflection, and a sense of belonging were rewarding experiences after overcoming cultural challenges.

*S3: To acquire well-being regardless of culture, I must be well-planned and prepared. First, I need to work hard to learn English and improve my language skills to communicate better with the locals. I also need to understand the culture, laws, and regulations of the United States to better adapt to the local environment. As long as I persevere and move forward courageously, I can achieve this goal and live a happy life in other countries.*

The category Cultural adaptations contain only one element: Motivation. Figure 4 shows that Motivation comprises three elements: Ability to please oneself, Personal goals and passion, and Academic achievement. The ability to please oneself represents the vital ability to appreciate one’s culture. Personal goals and passion represent those crucial elements in identifying purposeful goals, such as pursuing a specific career path and developing new skills. Most participants believed that understanding their desires could help them cope better with the stress of cultural adaptation, such as traveling worldwide, acquiring fluent oral English, and becoming interculturally competitive in other countries. Academic achievement encompasses three elements: Fulfilling dreams and aspirations, Working hard and being dedicated, and Success in education. Fulfilling dreams and aspirations was an essential motivation for participants. This involves seeking a higher education degree, embarking on a new career, and enhancing quality of life. Besides, in the participants’ opinion, hard work and dedication were effective methods to achieve cultural well-being and gain control of life. Success in education represents learning and thriving in a new cultural environment.

*S9: Passing the college entrance examination was a personal achievement for me and a step towards promoting cultural communication through art. It has equipped me with the necessary skills and knowledge to contribute to cultural development through public art. By injecting vitality into urban spaces, I hope to bridge cultural gaps and foster a deeper appreciation for diversity. Furthermore, bringing joy and inspiration to others through art makes me feel that I have a valuable and meaningful life, which is also a kind of indescribable happiness.*

Figure 4 also shows a new attribute of cultural intelligence: Globalization and responsibilities, which encompass Achieving common prosperity, Educational promotion, Enhancing social justice, Environmental issues, Global aging population, Network security, and Wealth inequality. The international issues that concern the participants represent how individuals consider these global challenges. Achieving common prosperity demonstrates the participants’ wish to create a more inclusive and equitable society where all individuals can thrive. Educational promotion, Enhancing social justice, and Wealth inequality highlight empathy and understanding toward others. Global aging population and Network security are two pressing concerns in today’s interconnected world, reflecting participants’ views on the importance of aging people’s well-being and a more secure and supportive global environment for all.

Furthermore, identity is commonly explored within social and intercultural frameworks. However, individuals’ perceptions of their identities extend far beyond these contexts. Kunchamboo et al. (2021) propose that research on identity should also consider individuals’ cognition, emotions, and behaviors toward the natural environment. They applied “ecological identity” to explain the dynamic process of how individuals interact with the natural environment, social roles, language, and self-reflection. Developing ecological identity requires attention to three key aspects: “Connectedness, care, and commitment.” These three aspects explain emotions, cognition, concern, responsibility, and obligation toward the natural environment, which are also important elements of well-being. As Figure 4 illustrates, Environmental issues involve some of the most pressing global challenges with far-reaching impacts, which include Carbon emissions, Energy conservation, Global warming, Marine ecosystem, Recycling, and Water pollution. These environmental problems are complex and interconnected, and the participants’ perspectives reflect a profound comprehension of global ecological cultures.

The last category of the cultural intelligence theme, as Figure 4 shows, is Seeking evidence-based reasoning, which includes Comparison, Comprehensive skills, Cross-cultural perspectives, Evidence-based interpretations, Knowledge review, and Seizing of every opportunity. These elements involve the participants’ strategies and approaches to dealing with cultural problems, representing metacognitive, cognitive, and behavioral CQ. Comparison and comprehensive skills help participants analyze cultural differences, embrace various viewpoints, and stay updated on cultural trends and developments. Cross-cultural perspectives and evidence-based interpretations help individuals better understand and empathize with others. Knowledge review and seizing every opportunity involve engaging with individuals from different cultural backgrounds and learning from their experiences without stereotypes or assumptions.

*S16*: *The concept of “well-being” in Chinese culture and Western culture emphasizes harmony and balance between individuals and society and considers it one of the most important goals in life. Happiness in both cultures is closely related to family, interpersonal relationships, health, and inner satisfaction. In both Chinese and Western cultures, people believe that spending quality time with family and friends is a part of well-being.*

##### 4.2.2.3 The theme of critical thinking

As Figure 5 shows, the theme of critical thinking involves three categories: Critical reflection and analysis, Mindfulness and meditation, and Open-mindedness. Applying critical thinking is a complex and multifaceted process requiring individuals to reflect critically and analyze, practice mindfulness and meditation, and cultivate open-mindedness.

Critical reflection and analysis consist of three elements that are essential for personal growth, as Figure 5 illustrates: Family values, Dealing with stereotypes, and Wisdom. This section demonstrates how participants addressed stereotypes, examined family values, and applied their wisdom to understand themselves and others better.

*S1: Although there are many differences between Chinese and Western cultures, there are many similarities in the concept of “happiness.” These similarities transcend cultural differences and become one of the common goals pursued by humanity.*

*S19: Christmas and the Spring Festival can be small consolations of “happiness” in Chinese and Western cultures. During Christmas, people hold Christmas parties and send greetings to family and friends, while Chinese people set off firecrackers and visit relatives during the Spring Festival. Both are expressions of the same emotions, so the “well-being” in Chinese and Western cultures embodies the same emotions of people.*

As Figure 5 demonstrates, Mindfulness and meditation contain only one element: Positive mindset, which involves a variety of personalities and habits that help participants overcome the challenges of achieving well-being: Inner self-improvement, Emotional stability, Inner peace, Adaptability to challenges, Inner strength and confidence, Self-management, Purposeful life, Fulfilling potential, and Being creative.

Mindfulness is associated with meditation, which involves noticing or ignoring rules, regulations, relationships, consequences, and contexts (Dacey et al., 2016). Ting-Toomey (2015) stated that in both Eastern and Western cultures, mindfulness is a way to cultivate awareness, presence, and empathy in communication and relationships. Holland et al. (2017) stated that cognitive function could be enhanced through mindfulness and meditation practices to improve critical thinking skills. Mindfulness and meditation can also help individuals reduce stress and negative emotions and cultivate a sense of adaptability to cultural identity negotiations (Ting-Toomey, 2015). The research findings in Figure 5 show how participants learn to approach cultural challenges to achieve personal growth. Creative thinking allows students to find innovative solutions to problems and navigate through the complexities of cultural adaptation. Stable emotions and inner peace also help them establish inner strength and confidence, enabling them to face difficulties with courage and resilience. Purposeful life and self-management empower participants to take control of their lives and manage their thoughts, emotions, and actions when dealing with cultural conflict.

*S12: As a human being, your life is filled with endless possibilities - choices that can lead to opportunities and opportunities that can lead to growth. However, if there is one task considered to be the most challenging in our lives, it is to become a true individual who thinks independently and knows themselves. We live in a complex and ever-changing world, surrounded by* var*ious expectations, values, and social pressures of different cultures. As members of society, we are inevitably influenced by external factors, but becoming a person means bravely facing our inner challenges and exploring our beliefs, values, needs, and goals.*

Moreover, Figure 5 demonstrates that Inner self-improvement includes Embracing artistic talents, Continuous learning and curiosity, Seeking critical thinking, Respecting inner desires, Navigating internal challenges, and Improving skills. Inner self-improvement provides a new perspective on self-growth. Participants thought that achieving cultural well-being was a process of learning and practicing; it required dedication and commitment to improve oneself and constantly striving for personal growth. Continuous learning and curiosity, improved skills, and critical thinking are the three essential components of a positive mindset, which enable individuals to expand their horizons and gain a deeper understanding of the world around them. Additionally, embracing artistic talent, navigating internal challenges, and respecting inner desires are three components that represent a significant intrinsic motivation driving identity formation. According to Waterman (2004), intrinsic motivation is an essential driving force that stimulates individuals’ strong interest in and passion for specific goals, values, and beliefs, thereby promoting identity development.

Figure 5 shows that the third category is Open-mindedness, which includes three elements: Accepting different cultural perspectives, Being flexible and adaptable, and Tolerance and compassion. Open-mindedness is essential for individuals to understand and appreciate various cultures. The results showed that participants learned to view well-being in different cultures from a tolerant and objective perspective. Tolerance and compassion are essential for individuals to deal with problems of culture and identity in various situations, such as undertaking global responsibility (Kunchamboo et al., 2021), coping with language learning barriers (Oxford, 2018), and facing human suffering (Ryff, 2022, 2023a, 2023b). This is the foundation for accepting different cultural perspectives, which creates a third space between cultures, as Bhabha (1990) proposed, allowing for a more fluid and flexible expression of cultural identity and avoiding cultural stereotypes.

##### 4.2.2.4 The theme of facing challenges

**Figure 6:**
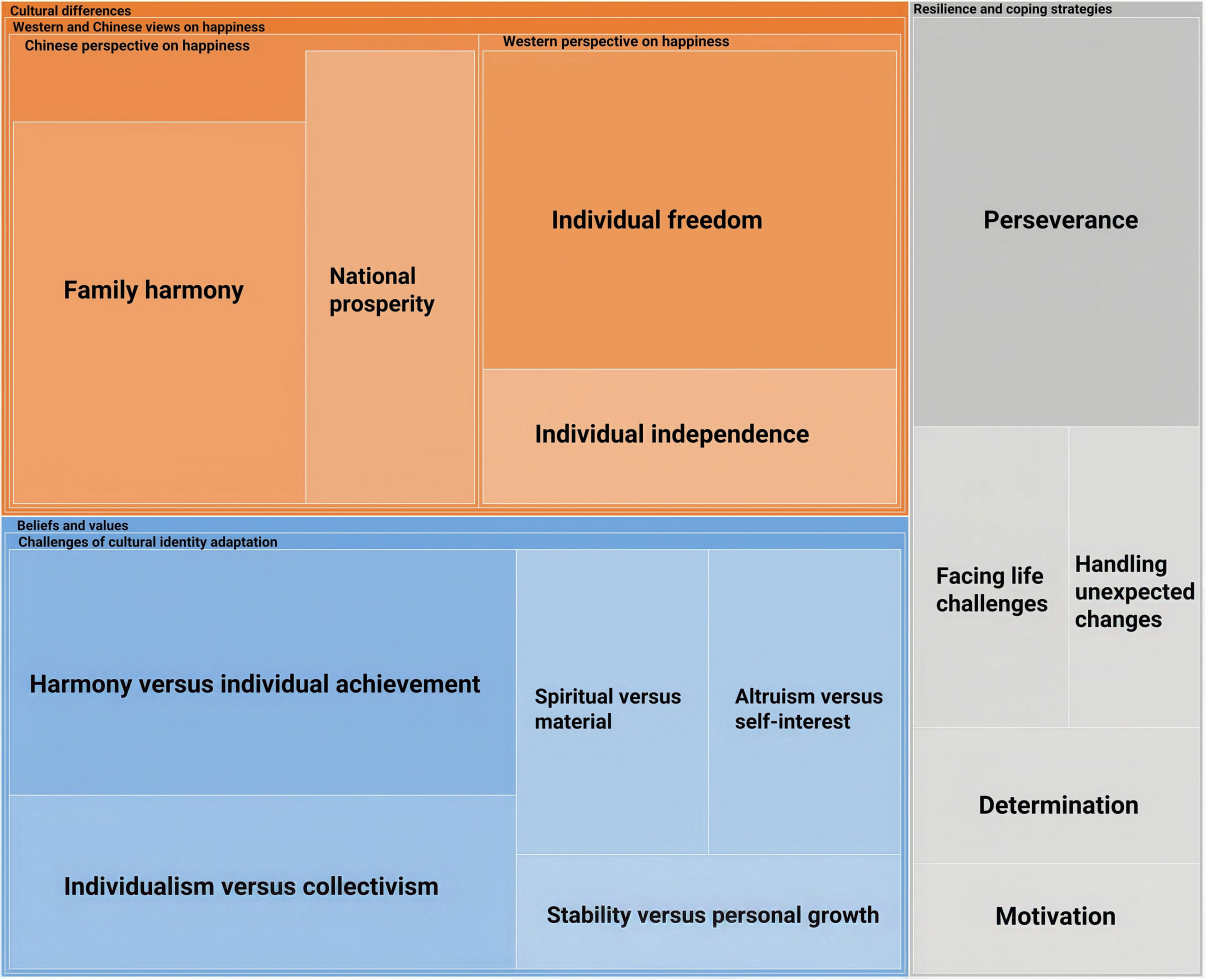
The theme of facing challenges.

Figure 6 shows that Facing challenges includes three categories: Cultural differences, Beliefs and values, Resilience and coping strategies.

The category of Cultural differences contains one element: Western and Chinese views on happiness, which can be divided into two attributes: Chinese perspective on happiness and Western perspective on happiness. In the participants’ views, the Chinese emphasize family harmony and the greater good, while the Westerners pursue individual independence and freedom. These differences are explicitly distinguished by beliefs and values that significantly impact cultural identity.

Beliefs and values involve one element: Challenges of cultural identity adaptation, which include Harmony versus individual achievement, Individualism versus collectivism, Spiritual versus material, Altruism versus self-interest, and Stability versus personal growth. This section briefly discusses the struggles between different beliefs and values, revealing that individuals inevitably navigate a complex web of beliefs and values to achieve well-being, each with their own conflicts. Participants expressed various thoughts about coping with a purposeful life in Chinese and Western cultures. Harmony versus individual achievement represents adapting to one’s cultural identity. In the participants’ minds, Chinese culture prioritizes group well-being and strives for harmony and cooperation. By contrast, Western cultures value individual achievement and personal success. Individualism versus collectivism demonstrates participants’ opinion that Western cultures emphasize the importance of individual freedom for individualism, while Chinese sometimes prioritize the group’s needs over those of the individual for collectivism. Altruism versus self-interest highlights the contrast between the inherent inclination to benefit others and the natural tendency to prioritize personal needs.

*S7: Nietzsche challenged traditional concepts of well-being, believing that happiness does not come from the pursuit of morality and reason but from individual self-realization and creativity. He advocated for people to break free from the constraints of traditional moral and value concepts, pursue their desires and passions, and thus achieve their value and happiness. Unlike more extroverted Westerners, Chinese people have traditionally been introverted, not overly enthusiastic in pursuing personal well-being, and often prioritizing the nation and society.*

*S11: Western culture emphasizes individualism, individual independence, freedom of choice, and pursuit of personal goals. In this culture, individuals emphasize their uniqueness, self-fulfillment, and the value of personal achievement. In Chinese culture, collectivism often influences self-worth, emphasizing the close relationship between individuals and the community. In collectivist cultures, individuals who maintain good social and family relationships frequently receive more support and security, increasing their happiness. Conversely, in individualistic cultures, individuals pursue independence and personal achievement and achieving personal goals can contribute to happiness.*

Moreover, Spiritual versus material represents the tension between material wealth and spiritual values in cultural identity adaptation. In participants’ minds, the Chinese prioritize spiritual fulfillment and inner peace, whereas in Western cultures, material wealth and possessions are highly emphasized and associated with psychological well-being. The participants thought it was necessary to balance these two values in this era of globalization. Additionally, Stability versus personal growth reveals that Chinese and Western cultures usually create conflicts between personal freedom and family bonds. Participants thought that the Chinese always valued stability and tradition, believing that changes represent uncertainty that might cause harmful consequences; on the contrary, in Western cultures, embracing change and adventure is part of life.

*S6: In Chinese culture, “well-being” is having a stable career, income, stable family, stable everything, a harmonious relationship among family members, and a deep love between them, always pursuing a stable life. In Western culture, “well-being” is a kind of spiritual satisfaction with unwavering love, steadfast friendship, and deep family ties. However, in Chinese culture, “happiness” feels more like a whole, as if as long as there is this whole, one will be happy, while in the West, it is more about a kind of happiness in one’s spirit.*

*S19: As Confucius said, the wise find joy in water, and the benevolent find joy in mountains. The wise are active, the benevolent are calm. The wise find joy, the benevolent find longevity. However, in Western culture, people usually pursue happiness through career success and material wealth. In traditional Chinese culture, pursuing happiness tends to be more inward, focusing more on moral cultivation and emotional communication.*

Figure 6 shows that Resilience and coping strategies comprise six elements: Perseverance, Facing life challenges, Handling unexpected changes, Determination, and Motivation. Cultural identity adaptation challenges communication, understanding, and acceptance of diverse perspectives (Ting-Toomey, 2015). Resilience and coping strategies reveal how participants navigate and overcome these challenges.

Perseverance and Facing life challenges are critical factors for individuals to maintain their goals and continue working toward them despite adversity. Participants’ narratives demonstrate they have overcome many unexpended challenges in acknowledging themselves in life challenges. Handling unexpected changes is a significant aspect of cultural identity adaptation, representing a willingness to embrace change and be flexible in one’s approach to navigating cultural differences (Dolce et al., 2023). Determination is essential when dealing with cultural identity adaptations. It allows individuals to overcome obstacles and setbacks and remain focused on their goals despite challenges (Waterman, 2011; Waterman and Schwartz, 2024; Waterman et al., 2024). Furthermore, as Figures 3–5 demonstrate, motivation is essential for cultural well-being. Figure 6 shows that motivation drives individuals to learn, develop, and engage with others. Sources of motivation can vary, stemming from personal aspirations, interpersonal connections, or a genuine curiosity about the world.

*S14: My most challenging goal by far is to climb the highest peak of Mount Sangguniang, which is very high and cold, with a harsh environment and thin oxygen. One slight mistake could lead to a lack of oxygen or physical exhaustion and death. However, it is beautiful there. Once you reach the top, you will feel pride and accomplishment. I currently do not have enough experience to challenge my highest peak, and I need to ensure my safety in life, so I am still in the preparation stage. This is the most challenging goal I have ever faced so far.*

## 5 Discussion

This research provides a holistic perspective on how individuals with critical thinking beliefs and cultural intelligence achieve their well-being when dealing with identity development challenges. As McAdams and Zapata-Gietl (2015) proposed, in Erikson’s theory of identity, individuals’ sense of identity over time is shaped by their experiences, abilities, and social roles. By understanding how these factors interact and influence one’s sense of self, individuals can develop a stronger sense of purpose and direction. From this perspective, identity development is the intricate process in which individuals comprehensively understand their qualities, values, and beliefs. This research shows that achieving well-being is not a goal isolated from the other aspects of life; it is intimately intertwined with one’s cultural identity. When individuals possess critical thinking beliefs and cultural intelligence, they navigate the challenges of identity development with greater resilience and adaptability.

Critical thinking and cultural intelligence play essential roles in cultural identity formation. In the quantitative part of this research, the CriTT (Critical Thinking Toolkit; Stupple et al., 2017), CQS (Cultural Intelligence Scale; Ang et al., 2007), the SFCQ (Short Form of Cultural Intelligence; Thomas et al., 2015), and the QEWB (Questionnaire for Eudaimonic Well-Being; Waterman et al., 2010) are applied to discover the interplay between critical thinking beliefs, cultural intelligence, and eudaimonic well-being. In Study 1, the SFCQ and CriTT were used to discover the correlations between three dimensions of CriTT (Confidence in Critical Thinking, Valuing Critical Thinking, Misconceptions about critical thinking) and three of SFCQ (Cultural Knowledge, Cultural Skills, and Cultural Metacognition). In the quantitative part of Study 2, CriTT, QEWB, and CQS were applied to investigate the eudaimonic well-being, three dimensions of CriTT, and four of CQS (Metacognitive CQ, Cognitive CQ, Motivational CQ, and Behavioral CQ). The research findings demonstrate the significant correlation between eudaimonic well-being, critical thinking beliefs, and cultural intelligence. Critical thinking helps individuals maintain an open and flexible attitude when facing cultural differences, enhancing their understanding and appreciation of diverse cultures. Cultural intelligence emphasizes individuals’ adaptability in different cultural contexts. They are crucial to personal growth and identity development, enabling individuals to think more deeply, reason more effectively, and ultimately lead a more prosperous life while pursuing well-being. The positive attitudes and beliefs about critical thinking enable individuals to question societal expectations, allowing them to forge their path rather than conform to predetermined roles.

In the qualitative part of Study 2, Figure 2 illustrates the identity landscape of participants’ perceptions, strategies, and comprehension of psychological well-being across cultures. Taking this stance, the concept of “cultures” within cultural identity evolves into a more multidimensional paradigm. Cultural differences can impact how individuals perceive themselves and their abilities to control their behaviors (McAdams, 2013). According to McAdams (2013), psychological selfhood is a framework consisting of “social actor, motivated agent, and autobiographical author.” “Actor” pertains to the representation of characteristics, personality, and other aspects of one’s identity that are shaped by continuous interactions with society. “Agent” refers to “personal goals, motives, values, hopes and fears, and other features” for realizing achievement in life span. “Author” refers to being the creator of “a meaningful narrative for life.” McAdams (2013) states that the effect of “author,” which is how individuals make sense of past, present, and future through narrative, significantly impacts identity formation. In this “social actor, motivated agent, and autobiographical author” framework, “author” is expected to bear the most significant and enduring cultural effects. The research findings in Study 2 demonstrate the map of well-being between cultures and present how these three psychological selfhoods (social actor, motivated agent, and autobiographical author) interacted with participants’ comprehension of cultural differences.

Purpose-building in life is influenced by various socio-cultural factors like language, societal norms, history, and belief systems (Moran, 2020). The four themes of Psychological well-being, Cultural intelligence, Critical thinking, and Facing challenges demonstrate how individuals approach identity constructions and pursue purpose in their lives. Participants’ narrations are mainly about how they executive the psychological selfhoods of “actor, agent, and author” (McAdams,2013) to realize their desired well-being. In the theme of well-being, Figure 3 illustrates the interconnection between how individuals cope with the relationship between the world and their internal selves. They have goals about the ideal life, such as personal growth, social relationships, and preferred lifestyle. In the theme of cultural intelligence, Figure 4 demonstrates the intricate web of cultural intelligence and its profound impact on individuals’ life purpose-building processes. Participants in the study described the challenges and strategies in coping with the cultural differences, which catalyze self-reflection and personal growth, leading them to redefine their goals and aspirations. This highlights cultural intelligence’s role in enriching individuals’ lives and deepening their understanding of themselves and the world. In the theme of critical thinking, Figure 5 illustrates the cognitive processes involved in purpose-building. Participants questioned and evaluated their values and beliefs in light of their experiences and interactions with the world around them. The results of this part reveal how individuals actively construct a purposeful life that is meaningful and authentic to them rather than passively accepting the purposes dictated by their culture or society. In the theme of facing challenges, Figure 6 shows that dealing with obstacles in life is a part of establishing psychological well-being. Despite their adversity, participants demonstrated resilience and perseverance in navigating these challenges. Many described how overcoming obstacles strengthened their sense of purpose and deepened their commitment to their goals. The research findings of this part highlight how facing challenges is essential for meaningful purpose-building and personal growth. By directly addressing obstacles and adapting to adversity, individuals can gain deeper self-awareness, achieve identity development, and lead more purposeful lives.

Besides, the results indicate that this research revealed how these attributes influence cultural identity development through critical thinking, cultural intelligence, and eudaimonia well-being. By organizing and interpreting personal experiences, the qualitative analysis in Study 2 effectively explores the complex map encompassing identity development, psychological well-being, critical thinking, and cultural intelligence. Together with the quantitative analysis, qualitative analysis offers a multidimensional view of well-being. Participants who exhibit high levels of eudaimonic well-being may demonstrate a harmonious integration of self-construction and self-discovery. These individuals may experience a profound sense of purpose and fulfillment grounded in a deep understanding of their identity. Psychological well-being encompasses sociality, reasoning, friendship, justice, good governance, practical wisdom, scientific knowledge, and contemplative wisdom (Fowers, 2016). Some scholars propose that happiness is influenced by three factors: set point, contextual factors, and intentional activities, with environmental factors also playing a role in individual well-being (Diener et al., 2003; Sheldon and Lyubomirsky, 2004). However, Waterman (2008)claims that psychological well-being is not limited to specific individuals but accessible to all. Modern eudaimonist philosophy adopts a broader perspective than Aristotle, which includes subjective components in defining eudaimonia. The subjective initiative is achieved through efforts to develop skills and talents and pursue worthwhile goals in life. Although individuals’ subjective initiative might be affected by external factors, attaining well-being through personal growth is also possible.

Being self is a challenging task in life’s journey. Psychological well-being theories provide us a critical perspective for an essential question of that journey: What kind of self should we be? Ryff’s (1989, 2014) multidimensional model emphasizes the significance of self-acceptance, positive relations, autonomy, environmental mastery, life purpose, and personal growth. In this research, cultural identity serves as a lens through which these dimensions are shaped. Furthermore, based on the eudaimonic identity theory, Waterman and Schwartz (2024) further develop the Integrative Theory of Intrinsic Motivation, which is constructed personal characteristics with self-determination, balance of challenges and skills, self-realization values, and engaged performance. These four characteristics encompass essential activities that aid in overcoming obstacles and challenges related to identity development: selection, evaluation, and ambition (Waterman and Schwartz,2024). From this perspective, individuals who value critical thinking and cultural intelligence will intentionally achieve psychological well-being through purposeful practice, cultivation, and reflection. The findings of this research serve as a source of inspiration for us to thrive and lead fulfilling lives, echoing the sentiment expressed by the esteemed Chinese artist Li Shutong: “见天地,见众生,见自己,” which can be interpreted as “understanding the world, empathizing with others, and introspecting on oneself.”

## 6 Limitation and future work

This research explores the intricate relationship between critical thinking beliefs, cultural intelligence, and psychological well-being within the context of cultural identity development. While the study provides valuable insights into the experiences of Chinese students, there is a need for ongoing research that continues to explore the intersection of culture, education, and student outcomes. In future research, it will be essential to address several limitations to enhance further our understanding of the relationship between critical thinking beliefs, cultural intelligence, and psychological well-being within cultural identity development. Firstly, longitudinal studies should be considered in future studies. Tracking participants’ development across various life stages and cultural contexts could illuminate the long-term effects of critical thinking and cultural intelligence on identity formation and psychological well-being. Secondly, the diversity of the sample in the current study may only partially represent a part of the population. The samples only comprised Chinese arts students, and this limitation of the cross-subjects and cross-cultures impacted the generalizability of the findings. Future research should include more diverse populations across different cultural backgrounds and academic disciplines to provide a comprehensive understanding of the relationship between critical thinking, cultural intelligence, and psychological well-being. Thirdly, the current study primarily focused on individual-level factors. Future research could explore how societal or institutional factors influence critical thinking, cultural intelligence, and well-being on a broader scale. By addressing these limitations, future research can contribute to exploring personal growth and fulfillment across diverse cultural contexts.

## Data Availability

The original contributions presented in the study are included in the article/supplementary material, further inquiries can be directed to the corresponding author.
